# Towards powerful experimental and statistical approaches to study intraindividual variability in labile traits

**DOI:** 10.1098/rsos.160352

**Published:** 2016-10-26

**Authors:** David J. Mitchell, Benjamin G. Fanson, Christa Beckmann, Peter A. Biro

**Affiliations:** Centre for Integrative Ecology, School of Life and Environmental Science, Deakin University, Geelong, Victoria 3216Australia

**Keywords:** behavioural predictability, behavioural plasticity, residual model, repeatability, behavioural syndrome, multiple burst

## Abstract

There is a long-standing interest in behavioural ecology, exploring the causes and correlates of consistent individual differences in mean behavioural traits (‘personality’) and the response to the environment (‘plasticity’). Recently, it has been observed that individuals also consistently differ in their residual intraindividual variability (rIIV). This variation will probably have broad biological and methodological implications to the study of trait variation in labile traits, such as behaviour and physiology, though we currently need studies to quantify variation in rIIV, using more standardized and powerful methodology. Focusing on activity rates in guppies (*Poecilia reticulata*), we provide a model example, from sampling design to data analysis, in how to quantify rIIV in labile traits. Building on the doubly hierarchical generalized linear model recently used to quantify individual differences in rIIV, we extend the model to evaluate the covariance between individual mean values and their rIIV. After accounting for time-related change in behaviour, our guppies substantially differed in rIIV, and it was the active individuals that tended to be more consistent (lower rIIV). We provide annotated data analysis code to implement these complex models, and discuss how to further generalize the model to evaluate covariances with other aspects of phenotypic variation.

## 1. Introduction

Ecologists and evolutionary biologists are often interested in studying the variation among individuals within a population and the consistency of these differences, particularly in the study of labile traits. To do so requires resampling individuals and assessing the relative proportion of overall variance that can be attributed to individual differences, most often quantified using ‘repeatability’ to estimate the consistency of scores through time [1–4]. Individuals often also vary in their response to their internal or external environment (‘plasticity’), and thus vary in temporal trends and/or responses to obvious contextual variation (e.g. [5,6], reviewed in [7]). However, even after accounting for both these hierarchical levels of systematic variation, considerable residual variation remains in labile traits (hereafter termed ‘residual intraindividual variability (rIIV)’; [8]), which can conceal important biological processes [9].

Behaviour is a particularly labile trait, which varies at multiple phenotypic levels, and is flexible in response to a changing environment. It has long been appreciated that individuals commonly differ in their mean level behaviour (often termed ‘personality’), and there is broad consensus that behaviour is, in general, repeatable [2]. However, the repeatability of behaviour is low on average, in that the majority of behavioural variation occurs within individuals, rather than among individuals [2]. The same can be said for physiological traits, such as metabolism, which are similarly labile [3]. Thus, the challenge is to better understand factors that affect this large but understudied component of labile trait variation.

Recent studies indicate that this rIIV can also significantly differ between individuals [8]. These differences are frequently observed under highly controlled conditions and after accounting for systematic temporal and/or contextual plasticity among individuals [8,10,11]. Residual IIV is typically viewed as short-term unpredictable fluctuations in an individual's trait scores in a given context [7]. Although the few studies that exist are predominantly behavioural, there are recorded examples of individual differences in rIIV in hormone physiology [12] and cattle milk yields [13]. Interestingly, rIIV itself has been shown to be repeatable, in the sense that rIIV has some within-individual consistency across months under controlled laboratory conditions [10] and across seasons in the field [14]. Furthermore, rIIV in non-behavioural traits and residual intragenotypic variability have heritable components [13,15] which demonstrates rIIV could be viewed as a trait that could be selected upon.

The study of individual differences in rIIV is clearly still in an exploratory stage, and we need more research to quantify rIIV effectively and to start building a consensus on the magnitude and correlates of rIIV. In turn, this will guide empirical and theoretical research into its proximate causes and ultimate functions. One reason this topic is rarely addressed, at least in part, is due to the large sample sizes required at both the within-individual level (repeated measures per individual) and between-individual levels (replicate individuals) to estimate effect sizes with precision [16]. Another possible reason this topic is relatively unexplored is the complexity of the analyses needed to quantify these levels of variation in the best possible way [11,16,17]. Under simple model scenarios, a statistically powerful analysis requires in the region of at least *N* = 50 individuals with 10 repeated measures [16]. To the best of our knowledge, no existing study has yet met even these conservative estimates of minimum sample sizes needed, and so we argue that rigorous estimates of the magnitude of variation in rIIV are lacking, thereby impeding progress on this topic. Ideally, such data need to also be measured in controlled conditions to negate the effect of unquantified contextual environmental variation that could affect some individuals more than others [8,9]. However, that is not to say we should not study rIIV in the field, but individual differences in rIIV will probably be hard to distinguish from responsiveness to unobserved sources of temporal or contextual variation [14,17,18].

Individual differences in rIIV, if widespread and substantial, have significant implications for the design of experiments and the statistical analysis of those data. These issues have only just begun to be noted from a design [8] and statistical perspective [16,19], and so the major aim of this study is to provide a model example of experimental design, sampling effort and data analysis to best study rIIV. To further our understanding of rIIV, studies should analyse the covariance of individual differences in rIIV with individual mean levels of that trait. Such correlations could help form predictions on how rIIV relates to better known axes of phenotypic variation, which may help to inform on the function or proximate causes of rIIV. There are some early indications that individual differences in rIIV can be related to individual mean trait values [8,14,20], but these correlations can be estimated more rigorously and powerfully using methods described below, compared with the two-step approach these studies employed. More generally, the presence of individual variation in rIIV indicates a violation of model assumptions that can potentially affect our inferences from any data of a labile trait. Indeed, a recent study demonstrated how ignoring this level of behavioural variation altered the biological conclusions drawn from the data, severely reducing the power of the study [18].

Instead of viewing rIIV as being due to unexplained stimuli and continuing to try to better control experiments, it may be better to view this variation as a trait in itself, on which evolution acts. It is thought that rIIV, in behaviour, may represent different ways to facilitate learning, social interactions or avoid predation. For example, hermit crabs on average exhibited higher latency to emerge from their shell and higher rIIV when exposed to predator cues [21], suggesting that high levels of rIIV could potentially reduce the predictability of prey behaviour to predators. In terms of learning, large declines in rIIV have been observed across adolescents in humans during a time of rapid learning and brain development [22]. Similarly, average rIIV in activity rates decreased over the course of months in mosquitofish when held in isolation [10]. Consequently, we could predict exploratory and reactive individuals to show higher rIIV to facilitate learning.

Here, we conducted a controlled laboratory study that quantified variation in activity rates at the among- and within-individual levels. We used *N* = 104 male guppies (*Poecilia reticulata*), and measured them approximately five times each per burst, across three distinct bursts of sampling, for a total of 15 repeated measures per individual. This level of sampling provides the most robust analysis and description of rIIV to date, yielding good precision in estimating among-individual variation in rIIV. In addition to providing a model example of burst sampling methods and experimental design required to rigorously study rIIV, we extend recent statistical methods [16] to assess whether individual differences in rIIV are related to individual predicted mean values. Our sampling design also permits us to estimate the repeatability of individual mean values over time, in addition to the familiar estimation of repeatability of individual scores (traditional repeatability or intraclass correlation). We also discuss how the statistical models we use can be further extended to assess how rIIV may covary with individual differences in plasticity, or how rIIV may covary with other aspects of phenotypic variation. We provide annotated code to assist those wishing to explore these questions on their own data.

## 2. Methods

### 2.1. Study species, housing, husbandry

We randomly selected 111 male guppies (*P. reticulata*) from laboratory stock. Guppies used were laboratory-raised descendants of fish collected from an invasive population in northern Queensland 4 years prior to this study (see [23] for more information). Data from sick fish were discarded, though data from fish which jumped out of the tank were retained in the analyses, and dead fish were replaced. Our final sample size was 104 individuals. The experiment was run in two batches of roughly 50 fish, separated by one week (and accounted for in our model, see equation (2.1)). A batch of fish was processed over 2 days and then left to recover and acclimate overnight before the behavioural trials began. Guppies were first anaesthetized with MS-222 (Sigma E10521, 0.2 g l^−1^, buffered to pH 7.6), gently dabbed with a Kimwipe to remove excess water, and weighed to the nearest 0.001 g. Fish were then placed individually in a 3 l tank with a 1 cm layer of gravel and an air-stone. Two tank walls were covered in opaque white plastic, so fish had no visual contact with neighbouring fish. A 4 cm square grid (5 wide, 3 high) was drawn on the back of the tank to aid observations of activity. Fish were fed once daily a drop from a pipette containing either finely crushed commercial flake food or twice weekly brine shrimp *nauplii,* and kept in a 12 L : 12 D photoperiod, consistent with the stock tanks. At day 9–10 post-processing, 50% of the water was changed, and positions of tanks were shuffled to control any position effect.

### 2.2. Behavioural measurements

To measure spontaneous activity, a single observer (D.J.M.) counted the number of lines crossed over a 2 min time period, from a distance of 1 m from the tank. Fish were deemed to have crossed a line when the head and pectoral fin had crossed. After assaying 25 fish, the temperature for each tank was measured to the nearest 0.1°C. Over the entire duration of the experiment, temperatures ranged from 23.8 to 25.6°C in a temperature-controlled laboratory. The range of temperatures an individual experienced across the experiment was minimal (median range = 0.65°C), and the range experienced within a week was lower still (median range = 0.2°C). Because subtle fluctuations in temperature can have a significant effect on the behaviour of ectotherms [5], we statistically controlled for temperature at the population level (see equation (2.1)).

### 2.3. Sampling design

Following recent suggestions, we employed a ‘burst’ sampling design [8,10]. These bouts of intensive data collection were taken over 2–3 days in each of three consecutive weeks (= three bursts per individual). Each individual was assayed on average five times per burst (range = 4–6). This form of burst sampling allows for the consistency of the mean and rIIV to be analysed through time [10,24], and allows for more efficient modelling of time-related change as it does not assume linear trends (details below). The first burst was run for two days after processing (Thursday–Friday), and bursts 2 and 3 were run for three days each (Wednesday–Friday). Activity was measured once in the morning (between 09.00 and 12.00) and once in the afternoon (between 13.00 and 16.00) on trial days. In total, we used data from 104 individuals with typically three bursts each and a total of 15–16 activity assays each (*N*_obs_ = 1477).

### 2.4. Statistical methods

Despite the potential importance of individual differences in rIIV to the study of labile traits, the requisite statistical tools have been introduced only recently to the field of ecology [16,17]. Previous analyses of rIIV involved either a two-step approach of fitting a model and extracting the residuals for use in a second analysis [8,10,21] or through fitting individual specific residual variances, without assuming a distribution [12,25]. However, such methods allow for only limited power and comparability between studies, whereas the doubly hierarchical GLM (DHGLM) offers a more parsimonious analysis of rIIV [16].

The DHGLM allows for the simultaneous analysis of a mean level model and a residual level model, the latter in log-linked standard deviations. Each is a linear model that can be specified to include both fixed and random effects, thus allowing one to ask whether individuals differ in their within-individual residual variances (rIIV). Such methods are explained in detail elsewhere [16]; therefore, the DHGLM will not be discussed at length here. However, further to the methods previously employed, we fit a random effect covariance matrix to analyse the correlation between individual variation in means and individual variation in rIIV. This covariance matrix can also be easily generalized, if desired, to evaluate whether the intercept and rIIV covary with individual variation in plasticity (random slope effects; details are given below).

The mean model (equation (2.1)) for the guppy data was fitted with the fixed effects of the batch (0 or 1), mass (continuous), time post-processing in weeks (integer), time of day (morning or afternoon) and temperature (continuous). The mean model also included the random intercept effects of individual identity (ID_j_) and burst nested within ID (equation (2.1)). The random intercept of ID creates a predicted value for the intercept of each ID_j_ (*j* = 1 : *N*_ID_). The nested random intercept effect of burst was created as a new dummy variable, representing the interaction between ID (*N*_ID_ = 104) and week (categorical; *N* = 3), creating a new factor (‘ID * Burst’; *N*_ID*Burst _= 294). A predicted ID * Burst deviation is then given from the predicted value of the random intercept of ID plus the predicted value for each ID * Burst_k_ (*k* = 1 : *N*_ID*Burst_), which is normally distributed (equation (2.4)).

The residual model (equation (2.2)) was fitted with the fixed effect of mass and time post-processing to test for effects of experience on rIIV, and a random intercept effect of ID, giving a predicted standard deviation (i.e. rIIV) for each ID_j_ ( *j* = 1 : *N*_ID_, equation (2.2)). Finally, we allowed for a covariance (equation (2.3)) between predicted mean values of activity (ID*_µj_*, equation (2.1)) and predicted log-standard deviation (${\mathrm{ID}}_{\sigma \varepsilon j}$, equation (2.2)) among individuals.

2.1 $$\begin{array}{rl} & \mu_{\mathrm{jk}}∼\beta_{0}+\beta_{1}\mathrm{Batch}+\beta_{2}\mathrm{Mass}+\beta_{3}\mathrm{Week}+\beta_{4}\mathrm{Temp}+\beta_{5}\mathrm{AMPM}+ID_{\mu j}+{\left(\mathrm{ID}*\mathrm{Burst}\right)}_{k}, \end{array}$$

2.2 $$\begin{array}{rl} & \mathrm{lo}g_{e}{\left(\sigma_{\varepsilon }\right)}_{j}∼\gamma_{0}+\gamma_{1}\mathrm{Mass}+\gamma_{2}\mathrm{Week}+ID_{\sigma_{\varepsilon }j}, \end{array}$$

2.3 IDμ,σj∼MVN(0,ΩID):ΩID=[σIDμ2 COVIDμ,IDσωIDσε2]

2.4 $$\begin{array}{rl} \text{and}\; & {\left(\mathrm{ID}*\mathrm{Burst}\right)}_{k}∼N(0,\sigma_{\mathrm{ID}*\mathrm{Burst}}^{2}). \end{array}$$

Activity rates were log-transformed to achieve normality and then *Z*-transformed (standardized to mean = 0, standard deviation = 1) to aid model fitting and to facilitate comparison of variance parameters. The variances of random intercepts in the mean model can, therefore, be interpreted as proportions of the total variances of the dataset. Mass and temperature were also *Z*-transformed to centre predictors and aid model fitting, whereas week was centred on the second week of trials. The covariance between the random intercepts in the mean and residual models can be highly sensitive to different transformations, and care must be taken when choosing a transformation [26]. In the case of our data, these were counts, which predict a covariation between the mean and variance proportional to the log-scale, hence we chose to first log-transform the data. Although we fit the data to a normal distribution opposed to a Poisson for statistical convenience, all assumptions of normality and linearity at each ID, ID*Burst and residual levels were checked *post hoc*, and all assumptions were satisfied. To further evaluate model performance, we also simulated data of known structure.

Data were analysed in the Bayesian, Markov Chain Monte Carlo software Stan [27], through the ‘RStan’ interface [28]. All parameters were given uninformative priors (see electronic supplementary material). Importantly, the Hamiltonian Monte Carlo sampler, employed by Stan, allows for an LKJ prior to the covariance of random effects [29], opposed to the inverse-Wishart priors typically used by the common Gibbs samplers. The inverse-Wishart priors may yield biases in estimations where the magnitude of variance parameters differs greatly, owing to the assumption of equal degrees of freedom across all dimensions in the covariance matrix [30]. We set the LKJ prior for the correlation to 2, equal to the number of dimensions in the matrix, which causes a small peak in the prior probability of independence of the random effects (COV_int,rIIV_ = 0). Three chains were run in parallel to evaluate convergence, for a posterior of 50 000 iterations with a 10 000 iteration warm-up. Plots of fitted values versus residuals were checked for goodness of fit as well as checks for normality at each of the levels of the analysis (predicted values of ID and ID*Burst). Stan and R code for data specification, the model and goodness-of-fit diagnostics are provided in electronic supplementary material; we also provide model code for the more commonly used JAGS program [27] to aid accessibility of such an analysis.

Through the addition of the ID*Burst interaction into the residual model, it is possible to assess the consistency of individual differences in rIIV through time, with methods analogous to those proposed to quantify the repeatability of the reaction norm intercept (equation (2.5), [24]). However, in quantifying the consistency of rIIV, it is important to have large numbers of repeated measures at the burst level. Owing to the relatively low number of repeats per burst (*N*_obs_ = 4–6), there was limited power to test for an effect of ID*Burst in the residual model, and inclusion was deemed to over-fit the model. We do, however, evaluate the consistency of mean values between individuals as the ‘repeatability’ of intercepts (*R*_int_; equation (2.5)). Variations in reaction norms indicate that mean differences may not be maintained through time, or across contexts [4,31]. Using reaction norms, one can calculate the correlation between trait means across contexts or time [31], and similarly, *R*_int_ (i.e. the ‘intraclass correlation coefficient’) quantifies the consistency of the mean values across bursts.

2.5 $$R_{\mathrm{int}}=\frac{\sigma_{\mathrm{ID}}^{2}}{\sigma_{\mathrm{ID}}^{2}+\;\sigma_{\mathrm{ID}*\mathrm{Burst}}^{2}}.$$

We also quantified the ‘short-term’ (conditional) repeatability (*sensu* [24]) which accounts for individual differences in time-related change. This repeatability, thus, quantifies the consistency of scores within the short timeframe of bursts (equation (2.6), [24]). We also estimated the ‘long-term’ (unconditional) repeatability (equation (2.7)), which, by contrast, estimates the repeatability of scores without accounting for time-related change [4], and gives the overall consistency of scores expressed across all bursts, for the duration of the study. Finally, as a standardized metric of among-individual variation in rIIV, we transformed the variance parameter in the residual model to the coefficient of variation in predictability (CV_P_, equation (2.8)), as prescribed by Cleasby *et al*. [16].

2.6 $$\begin{array}{rl} R_{\mathrm{short}\text{-}\mathrm{term}} & =\frac{\sigma_{\mathrm{ID}}^{2}+\sigma_{\mathrm{ID}*\mathrm{Burst}}^{2}}{\sigma_{\mathrm{ID}}^{2}+\sigma_{\mathrm{ID}*\mathrm{Burst}}^{2}+\exp{\left(\gamma_{0}\right)}^{2}}, \end{array}$$

2.7 $$\begin{array}{rl} R_{\mathrm{long}\text{-}\mathrm{term}} & =\frac{\sigma_{\mathrm{ID}}^{2}}{\sigma_{\mathrm{ID}}^{2}+\sigma_{\mathrm{ID}*\mathrm{Burst}}^{2}+\exp{\left(\gamma_{0}\right)}^{2}} \end{array}$$

2.8 $$\begin{array}{rl} \mathrm{and}\;CV_{P} & =\sqrt{\exp(\omega_{ID_{\sigma_{\varepsilon }}}^{2})-1}. \end{array}$$

To evaluate the importance of heterogeneous rIIV to the model fit, we ran a model with the random effect of ID removed from the residual model, and calculated the change in fit using Watanabe–Akaike information criterion (WAIC) with the ‘loo’ package [32].

## 3. Results

There was no overall effect of mass (*β*_2_ = −0.005, 95% CRI = [−0.12, 0.12]) or temperature (*β*_4_ = −0.05 [−0.12, 0.014]) on activity rates. Fish assayed in the first batch were more active than the second (*β*_1_ = −0.37 [−0.61, −0.13]). There was no directional effect of time across the three weeks (*β*_3_ = 0.029 [−0.051, 0.11]), and fish were more active in the afternoon trials than the morning (*β*_5_ = 0.068 [0.009, 0.13]). The intercept in the mean model (*β*_0_) was 0.14 [−0.034, 0.31].

**Figure 1.**
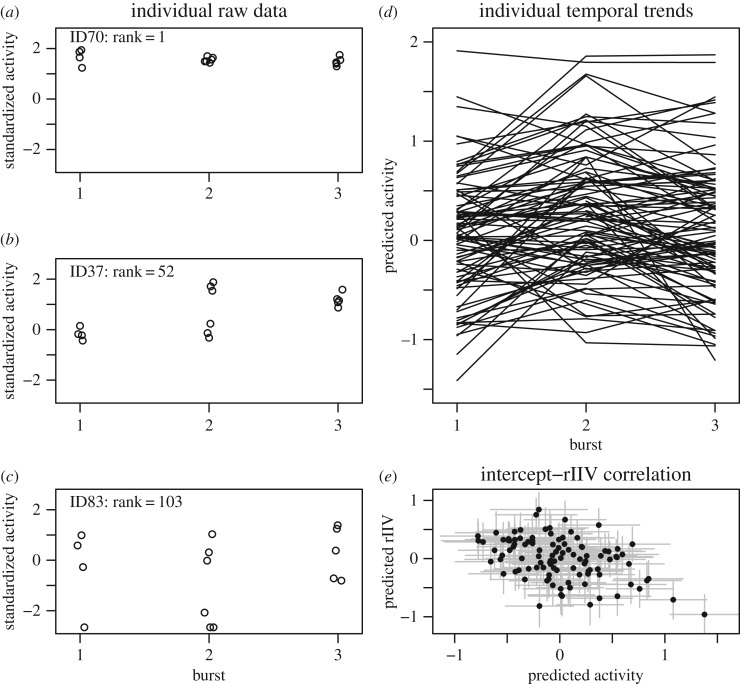
Displayed are the raw data for three example individuals across three bursts of observations, (*a*) shows the individual with the lowest rIIV, (*b*) shows the individual with the median estimate of rIIV and (*c*) shows the individual with the second highest rIIV. Panel (*d*) shows the temporal trends of all guppies across the three bursts. Panel (*e*) shows the correlation between the predicted mean intercepts and residual rIIV. Each point represents an individual, with the error bars representing one standard deviation of the credible distribution.

There was a substantial among-individual standard deviation in mean activity (random intercept: $\sigma_{ID_{\mu }}=0.51$ [0.41, 0.62]), and individuals also differed in their mean activity rates across bursts (random intercept: *σ*_ID*Burst_ = 0.46 [0.38, 0.55]), indicating that individuals differed in temporal patterns (figure 1*d*). After accounting for time-related change, a moderate amount of residual (within-burst) variation remained on average (mean residual standard deviation: exp(*γ*_0_) = 0.61 [0.55, 0.67]). It was evident that individual mean values were moderately consistent over time (*R*_int_ = 0.55 [0.39, 0.69]). In other words, there was evidence for the maintenance of individual differences in activity, a key aspect of personality, though there was a substantial degree of variation across bursts (figure 1*d*). The repeatability of scores while accounting for the effect of time-related change (conditional repeatability) was moderately high (*R*_short-term_ = 0.56 [0.48, 0.64]). By contrast, consistency of scores within individuals without taking account of time-related change was moderately low (*R*_long-term_* *= 0.31 [0.21, 0.41]).

Neither the fixed effects of mass (*γ*_1_ = −0.067 [−0.16, 0.025]) nor time (*γ*_2_ = −0.046 [−0.1, 0.011]) had an effect on the residual variation (rIIV). There was substantial among-individual variation in rIIV, and this was estimated with considerable precision, given the small credible distribution ($\omega_{ID_{\sigma }}=0.42$ [0.35, 0.5], figure 1*a*–*c*), giving a CV_P_ of 0.43 [0.36, 0.53]. Given the very large differences in rIIV between individuals, the effect of this term was highly important in the model (WAIC_Full_ = 3036, WAIC_Reduced_ = 3304). Individuals with larger rIIV also tended to be more sedentary on average (*r*_Int,rIIV_ = −0.34 [−0.56, −0.076], figure 1*e*).

## 4. Discussion

Here, we demonstrated consistent individual differences in the activity rates of guppies, across multiple time periods, and at several hierarchical levels of variation. First, we observed that individuals showed pronounced differences in their rIIV. These differences were evident under highly controlled conditions, after accounting for both significant and substantial individual differences in mean values and accounting for change in activity over time. While the model fit does not enable us to calculate the repeatability of rIIV *per se*, the burst sampling experimental design does allow for the rejection of the null hypothesis of no consistency in rIIV, which corroborates previous work, quantifying the repeatability of rIIV [10,14]. Had we collected even more data, we could have estimated the consistency of rIIV in the same way as we estimated the consistency of individual mean values over time (see equation (2.5)). To do so would require adding an ID*Burst term to the residual model and adding a second covariance matrix at the burst level.

We believe that our study demonstrates well how burst sampling offers a powerful approach to partition the variance among individuals, and within-individual variance explained by temporal trends, to appropriately control for these temporal patterns in behavioural data [4]. This approach revealed that individual mean values were moderately maintained over time (*R*_int_ = 0.55), a key assumption of animal personality, though with considerable fluctuations in mean values through time (figure 1*d*). Furthermore, examining the repeatability of activity scores revealed substantially higher within-burst consistency than across bursts (0.56 versus 0.31), further indicating the extent to which individuals differed in temporal trends [4]. This reduction in repeatability with time is consistent with results from meta-analyses of behavioural repeatability [2] and metabolic repeatability [3] which showed longer time intervals over which repeated measures are taken to reduce the repeatability of scores. Importantly, the multiple burst sampling design allows for modelling of time as a categorical variable, and therefore does not assume individual temporal trends to be linear (figure 1*d*), in contrast to more familiar random regression methods.

By quantifying the correlation between predicted mean level trait and rIIV among individuals, we can begin to evaluate whether rIIV relates to better known behavioural syndromes. There was a moderate negative correlation between mean activity rate and rIIV (figure 1*e*). As active individuals may be typically bolder, more aggressive and less responsive to environmental change [33–35], high rIIV could represent a more responsive phenotype and fit into the reactive syndrome, which predicts positive covariations between these traits and being fast explorers and more aggressive. Such a prediction would be consistent with a correlation between high rIIV and low boldness in hermit crabs [8]. Conversely, in a study on flight initiation distances, bolder agama lizards (*Agama planiceps*) were also more variable, potentially offsetting the risks associated with boldness by reducing predictability to the predator [14].

While there are biological reasons to predict a mean-variance covariance among individuals, one could also argue any such covariance to be an artefact of the scaling of the data, and another transformation would have yielded different results. This is because the log-transformation, as with other power function transformations (e.g. square-root) each predict a different positive relationship between the mean and variance. Such scaling issues could be addressed using methods such as a Box–Cox transformation whereby the data are raised to a series of power functions in search of the most likely fit to a normal distribution [16,26,36]. However, we argue that this is an unadvisable and arbitrary practice, which will further confuse the understanding of residual variation. These methods may not be reproducible in a replicated experiment, yielding different transformations, and the model will not account for this uncertainty in scaling. Quantifying the covariance between the mean intercept and rIIV will also allow for more critical and formal appraisal of the scaling of the data.

Our inclusion of a covariance matrix linking random effects in the mean and residual sides of the model is an important extension to the DHGLMs previously described and used in behavioural ecology [11,16,17]. Not only does this extension allow for quantifying the among-individuals correlation between the mean and rIIV in a more powerful and parsimonious way than the two-step process previously used [10,14,21], the covariance matrix can also be easily generalized to include reaction norms. Environments are often highly dynamic, with individuals responding to multiple environmental factors, which may interact in complex ways [6]. More environmentally responsive (i.e. more ‘plastic’) individuals could, therefore, have greater rIIV, owing to greater organismal error in calculating the ideal trait value from moment to moment [9,37,38]. Individuals may also be differentially constrained in the range of behavioural or physiological scores they can express, and their ability to gain knowledge on the current environment, which may also affect the amount of organismal error [37]. Limitations to an individual's ability to express a range of scores could limit both contextual plasticity and rIIV, while limiting the reliability of information increases rIIV. Theoretical models of plasticity based on Bayesian updating processes predict rIIV and residual intragenotypic variability to inform the prior distribution for the phenotypic scope for developmental plasticity [39], and these covariance matrices will prove useful in empirically testing these predictions. An important caution though, where individuals differ in both the magnitude and direction of their reaction norms, the most plastic individuals will be those with the largest positive and negative slopes, and the relationship between plasticity and reaction norms will not be a simple linear function [7].

Such a covariance matrix can also be specified to evaluate how rIIV relates to other aspects of the phenotypic variation. The matrix explaining the multivariate distribution of an individual phenotypic variation can link models with different response variables (phenotypic traits) at the individual level to assess how rIIV fits into better known axes of phenotypic variation (e.g. whether rIIV in activity correlates with boldness), and whether individuals are consistently variable across traits (e.g. whether high rIIV in activity correlates with high rIIV in boldness). However, as dimensionality in the covariance matrix increases, the positive definite assumption becomes more problematic in Bayesian models [30], and the recommended priors here will increase the prior probability of independence, making the analysis more conservative.

The social environment may be an important cue for individuals in the level of rIIV to express, and in this experiment, the effect of socially isolating individuals may have altered their mean behaviour and rIIV. This is because individual differences in rIIV can create differences in the predictability to predators or social partners. Where animals repeatedly interact, past experience informs later encounters [40], and being predictable can aid in social interactions where individuals aim to cooperate or coordinate behaviours [41]. Feedback between socially responsive and consistent individuals could maintain variation in both traits [42–44]. Conversely, in the short term, social interactions may homogenize behavioural variation among individuals and stabilize activity patterns to facilitate social functioning [41]. However, male guppies are much less social than females in this species [45], which may minimize this effect.

From comparisons with the psychology literature, one would predict rIIV to differ between learning types and to decrease with increasing experience and across ontogeny [39]. However, this did not appear to be the case in this experiment, and rIIV did not change with time over the three weeks of observations. In another short-lived Poeciliid (mosquitofish, *Gambusia holbrooki*), rIIV in activity rates decreased across the course of months, perhaps representing ontogenetic change or continued acclimation to being held in isolation [10]. Furthermore, as experience builds and animals acclimate (or habituate) to a new environment, a similar decrease in rIIV could be expected on a shorter timeframe, and such an effect is perhaps evident in the initial highly erratic and unpredictable responses of damselfish acclimating to the laboratory environment [46].

While the biological implications of individual differences in rIIV are at this stage speculative, the methodological implications are well established. The mixed-effect models typically used in the study of labile traits have the assumption of equal variances within-individuals. A comparison of individual predicted values taken from two standard deviations either side of the mean rIIV would yield a 28-fold difference in residual variances. This degree of heterogeneity would demonstrate a clear violation of statistical assumptions if ignored in the model, and highlights the importance of considering rIIV in the study of behavioural traits. Such variation should be visualized in the plots of raw data (figure 1*a*–*c*), and plotting out residuals by individuals is an important diagnostic step in the analysis of labile traits.

In conclusion, in this experiment, we have provided a model example of experimental design and analysis to study rIIV, and a highly robust analysis of individual differences in rIIV. Male guppies were shown to differ greatly in their residual variation, the consistency of mean values over time and consistency of scores over time that were (not surprisingly) dependent on whether or not we accounted for time-related change. We build on previously discussed statistical approaches [16] to evaluate how rIIV correlates with the mean trait, and discuss how to further extend the model to incorporate covariances between rIIV and plasticity (given by reaction norms). These additions will open novel avenues of research, and we suggest that future work should use these statistical methods and burst sampling strategies to test predictions of the causes and consequences of rIIV in behaviour.

## Supplementary Material

R code for doubly-hierarchical GLM in Stan

## Supplementary Material

JAGS Raw data

## Data accessibility

All data and data analysis code are available in the electronic supplementary material.

## References

[1] Wolak ME, Fairbairn DJ, Paulsen YR 2012 Guidelines for estimating repeatability. Methods Ecol. Evol. 3, 129–137. (doi:10.1111/j.2041-210X.2011.00125.x)

[2] Bell AM, Hankison SJ, Laskowski KL 2009 The repeatability of behaviour: a meta-analysis. Anim. Behav. 77, 771–783. (doi:10.1016/j.anbehav.2008.12.022) 24707058 10.1016/j.anbehav.2008.12.022 PMC3972767

[3] White CR, Schimpf NG, Cassey P 2013 The repeatability of metabolic rate declines with time. J. Exp. Biol. 216, 1763–1765. (doi:10.1242/jeb.076562) 23264481 10.1242/jeb.076562

[4] Biro PA, Stamps JA 2015 Using repeatability to study physiological and behavioural traits: ignore time-related change at your peril. Anim. Behav. 105, 223–230. (doi:10.1016/j.anbehav.2015.04.008)

[5] Biro PA, Beckmann C, Stamps JA 2010 Small within-day increases in temperature affects boldness and alters personality in coral reef fish. Proc. R. Soc. B 277, 71–77. (doi:10.1098/rspb.2009.1346) 10.1098/rspb.2009.1346 PMC2842624 19793748

[6] Westneat DF, Hatch MI, Wetzel DP, Ensminger AL 2011 Individual variation in parental care reaction norms: integration of personality and plasticity. Am. Nat. 178, 652–667. (doi:10.1086/662173) 22030734 10.1086/662173

[7] Stamps JA 2015 Individual differences in behavioural plasticities. Biol. Rev. 91, 534–567. (doi:10.1111/brv.12186) 25865135 10.1111/brv.12186

[8] Stamps JA, Briffa M, Biro PA 2012 Unpredictable animals: individual differences in intraindividual variability (IIV). Anim. Behav. 83, 1325–1334. (doi:10.1016/j.anbehav.2012.02.017)

[9] Westneat DF, Wright J, Dingemanse NJ 2014 The biology hidden inside residual within-individual phenotypic variation. Biol. Rev. 90, 729–743. (doi:10.1111/brv.12131) 25080034 10.1111/brv.12131

[10] Biro PA, Adriaenssens B 2013 Predictability as a personality trait: consistent differences in intraindividual behavioural variation. Am. Nat. 182, 621–629. (doi:10.1111/1365-2656.12210) 24107369 10.1086/673213

[11] Bridger D, Bonner SJ, Briffa M 2015 Individual quality and personality: bolder males are less fecund in the hermit crab *Pagurus bernhardus*. Proc. R. Soc. B 282, 20142492 (doi:10.1098/rspb.2014.2492) 10.1098/rspb.2014.2492 PMC4345441 25673676

[12] Montiglio PO, Garant D, Pelletier F, Réale D 2015 Intra-individual variability in fecal cortisol metabolites varies with lifetime exploration and reproductive life history in eastern chipmunks (*Tamias striatus*). Behav. Ecol. Sociobiol. 69, 1–11. (doi:10.1007/s00265-014-1812-x)

[13] Rönnegård L, Felleki M, Fikse WF, Mulder HA, Strandberg E 2013 Variance component and breeding value estimation for genetic heterogeneity of residual variance in Swedish Holstein dairy cattle. J. Dairy Sci. 96, 2627–2636. (doi:10.3168/jds.2012-6198) 23415533 10.3168/jds.2012-6198

[14] Highcock L, Carter AJ 2014 Intraindividual variability of boldness is repeatable across contexts in a wild lizard. PLoS ONE 9, e95179 (doi:10.1371/journal.pone.0095179) 24733271 10.1371/journal.pone.0095179 PMC3986370

[15] Ayroles JF, Buchanan SM, O'Leary C, Skutt-Kakaria K, Grenier JK, Clark AG, Hartl DL, de Bivort BL 2015 Behavioral idiosyncrasy reveals genetic control of phenotypic variability. Proc. Natl Acad. Sci USA 112, 6706–6711. (doi:10.1073/pnas.1503830112) 25953335 10.1073/pnas.1503830112 PMC4450409

[16] Cleasby IR, Nakagawa S, Schielzeth H 2015 Quantifying the predictability of behaviour: statistical approaches for the study of between-individual variation in the within-individual variance. Meth. Ecol. Evol. 6, 27–37. (doi:10.1111/2041-210X.12281)

[17] Westneat DF, Schofield M, Wright J 2013 Parental behavior exhibits among-individual variance, plasticity, and heterogeneous residual variance. Behav. Ecol. 24, 598–604. (doi:10.1093/beheco/ars207)

[18] Beckmann C, Biro PA, Martin K 2015 Hierarchical analysis of avian re-nesting behavior: mean, across-individual, and intra-individual responses. Behav. Ecol. Sociobiol. 69, 1631–1638. (doi:10.1007/s00265-015-1974-1)

[19] Cleasby IR, Nakagawa S 2011 Neglected biological patterns in the residuals. Behav. Ecol. Sociobiol. 65, 2361–2372. (doi:10.1007/s00265-011-1254-7)

[20] Jennings DJ, Hayden TJ, Gammell MP 2013 Personality and predictability in fallow deer fighting behaviour: the relationship with mating success. Anim. Behav. 86, 1041–1047. (doi:10.1016/j.anbehav.2013.09.009)

[21] Briffa M 2013 Plastic proteans: reduced predictability in the face of predation risk in hermit crabs. Biol. Lett. 9, 20130592 (doi:10.1098/rsbl.2013.0592) 23985348 10.1098/rsbl.2013.0592 PMC3971704

[22] MacDonald SW, Nyberg L, Bäckman L 2006 Intra-individual variability in behavior: links to brain structure, neurotransmission and neuronal activity. Trends Neurosci. 29, 474–480. (doi:10.1016/j.tins.2006.06.011) 16820224 10.1016/j.tins.2006.06.011

[23] Guevara-Fiore P 2012 Early social experience significantly affects sexual behaviour in male guppies. Anim. Behav. 84, 191–195. (doi:10.1016/j.anbehav.2012.04.031)

[24] Araya-Ajoy YG, Mathot KJ, Dingemanse NJ 2015 An approach to estimate short-term, long-term, and reaction norm repeatability. Methods Ecol. Evol. 6, 1462–1473. (doi:10.1111/2041-210X.12430)

[25] Briffa M, Bridger D, Biro PA 2013 How does temperature affect behaviour? Multilevel analysis of plasticity, personality and predictability in hermit crabs. Anim. Behav. 86, 47–54. (doi:10.1016/j.anbehav.2013.04.009)

[26] Yang YE, Christensen OF, Sorensen D 2011 Analysis of a genetically structured variance heterogeneity model using the Box–Cox transformation. Genet. Res. 93, 33–46. (doi:10.1017/S0016672310000418) 10.1017/S0016672310000418 21349235

[27] Stan Development Team. 2015 Stan: a C++ library for probability and sampling, version *2.8.0* (http://mc-stan.org)

[28] Stan Development Team. 2015 rstan: R interface to stan. 2.9 ed (http://mc-stan.org)

[29] Lewandowski D, Kurowicka D, Joe H 2009 Generating random correlation matrices based on vines and extended onion method. J. Multivariate Anal. 100, 1989–2001. (doi:10.1016/j.jmva.2009.04.008)

[30] Gelman A, Carlin JB, Stern HS, Rubin DB 2004 Bayesian data analysis. Boca Raton, FL: Chapman & Hall.

[31] Brommer JE 2013 Variation in plasticity of personality traits implies that the ranking of personality measures changes between environmental contexts: calculating the cross-environmental correlation. Behav. Ecol. Sociobiol. 67, 1709–1718. (doi:10.1007/s00265-013-1603-9)

[32] Vehtari A, Gelman A, Gabry J 2015 loo: efficient leave-one-out cross-validation and WAIC for Bayesian models. R package version 0.1.3. (https://github.com/jgabry/loo)

[33] Benus R, Koolhaas J, Van Oortmerssen G 1987 Individual differences in behavioural reaction to a changing environment in mice and rats. Behaviour 100, 105–122. (doi:10.1163/156853987X00099)

[34] Benus RF, Den Daas S, Koolhaas JM, Van Oortmerssen GA 1990 Routine formation and flexibility in social and non-social behaviour of aggressive and non-aggressive male mice. Behaviour 112, 176–193. (doi:10.1163/156853990X00185)

[35] Carere C, Drent P, Privitera L, Koolhaas J, Groothuis T 2005 Personalities in great tits, *Parus major*: stability and consistency. Anim. Behav. 70, 795–805. (doi:10.1016/j.anbehav.2005.01.003)

[36] Box GE, Cox DR 1964 An analysis of transformations. J. Roy. Stat. Soc. B. Met. 26, 211–252.

[37] DeWitt TJ, Sih A, Wilson DS 1998 Costs and limits of phenotypic plasticity. Trends Ecol. Evol. 13, 77–81. (doi:10.1016/S0169-5347(97)01274-3) 21238209 10.1016/s0169-5347(97)01274-3

[38] Tonsor SJ, Elnaccash TW, Scheiner SM 2013 Developmental instability is genetically correlated with phenotypic plasticity, constraining heritability, and fitness. Evolution 67, 2923–2935. (doi:10.1111/evo.12175) 24094343 10.1111/evo.12175

[39] Stamps JA, Krishnan V 2014 Combining information from ancestors and personal experiences to predict individual differences in developmental trajectories. Am. Nat. 184, 647–657. (doi:10.1086/678116) 25325748 10.1086/678116

[40] Milinski M, Külling D, Kettler R 1990 Tit for tat: sticklebacks (*Gasterosteus aculeatus*) ‘trusting’ a cooperating partner. Behav. Ecol. 1, 7–11. (doi:10.1093/beheco/1.1.7)

[41] Wolf M, Krause J 2014 Why personality differences matter for social functioning and social structure. Trends Ecol. Evol. 29, 306–309. (doi:10.1016/j.tree.2014.03.008) 24679987 10.1016/j.tree.2014.03.008

[42] Wolf M, Van Doorn GS, Weissing FJ 2008 Evolutionary emergence of responsive and unresponsive personalities. Proc. Natl Acad. Sci. USA 105, 15 825–15 830. (doi:10.1073/pnas.0805473105) 10.1073/pnas.0805473105 PMC2572984 18838685

[43] Laskowski KL, Bell AM 2013 Competition avoidance drives individual differences in response to a changing food resource in sticklebacks. Ecol. Lett. 16, 746–753. (doi:10.1111/ele.12105) 23489482 10.1111/ele.12105 PMC3972385

[44] Wolf MWG, Doorn SV, Weissing FJ 2011 On the coevolution of social responsiveness and behavioural consistency. Proc. R. Soc. B 278, 440–448. (doi:10.1098/rspb.2010.1051) 10.1098/rspb.2010.1051 PMC3013403 20739321

[45] Croft DP, Krause J, James R 2004 Social networks in the guppy (*Poecilia reticulata*). Proc. R. Soc. B 271 (Suppl. 6), S516–S519. (doi:10.1098/rsbl.2004.0206) 10.1098/rsbl.2004.0206 PMC1810091 15801620

[46] Biro PA 2012 Do rapid assays predict repeatability in labile (behavioural) traits? Anim. Behav. 83, 1295–1300. (doi:10.1016/j.anbehav.2012.01.036)

